# Relationship between therapeutic efficacy of doxorubicin-transferrin conjugate and expression of P-glycoprotein in chronic erythromyeloblastoid leukemia cells sensitive and resistant to doxorubicin

**DOI:** 10.1007/s13402-014-0205-5

**Published:** 2014-11-20

**Authors:** Marzena Szwed, Katarzyna D. Kania, Zofia Jozwiak

**Affiliations:** 1 Department of Thermobiology, Faculty of Biology and Environmental Protection, University of Lodz, Pomorska 141/143 Street, 90-236 Lodz, Poland; 2 Laboratory of Transcriptional Regulation, Institute for Medical Biology, PAS, 93-232 Lodz, Lodowa 106 street, Poland

**Keywords:** Doxorubicin, Transferrin, Drug carriers, Multidrug resistance, P-glycoprotein

## Abstract

**Background:**

Conjugation of anti-neoplastic agents with human proteins is a strategy to diminish the toxic side effects of anthracycline antibiotics. We have developed a novel doxorubicin-transferrin (DOX-TRF) conjugate aimed to direct anticancer drugs against therapeutic targets that display altered levels of expression in malignant versus normal cells. Our previous work has shown that the cellular bio-distribution of the conjugate is dependent on a dynamic balance between influx and efflux processes. Here, we set out to investigate whether P-glycoprotein (P-gp) expression may affect DOX-TRF conjugate-induced cellular drug accumulation and cytotoxicity.

**Results:**

All experiments were carried out on human erythromyeloblastoid cells exhibiting P-gp over-expression (K562/DOX) and its drug sensitive parental line (K562). MTT cytotoxicity, flow cytometry, fluorescence microscopy and RT-PCR assessments revealed that the investigated conjugate (DOX-TRF) possesses a greater cytotoxic potential than free DOX.

**Conclusion:**

Our data suggest that the newly developed DOX-TRF conjugate is a less P-gp dependent substrate than free DOX and, consequently, may be used in a clinical setting to increase treatment efficacy in resistant human tumors.

## Introduction

Anthracyclines represent an important class of anti-neoplastic agents that are active against a variety of hematologic [1, 2] and solid [3, 4] tumors. However, the clinical effectiveness of anthracyclines is often restricted by dose-limiting cardiac toxicity and myelosuppression [5]. The toxicity of doxorubicin (DOX), which belongs to these antrachinone antibiotics, is connected to the apoptosis of cancer cells by a complex network of events, which includes intercalation into DNA, generation of reactive oxygen species, topoisomerase II inhibition and DNA damage [6]. However, successful anticancer chemotherapy through the application of DOX requires a sufficient number of molecules enabling DOX to be delivered to the neoplastic cells while sparing normal cells. Attaching anthracycline antibiotic polymers, and especially tumor-associated antigens, has long been envisioned as a means to more selectively delivering chemotherapeutic agents [7]. Thus, DOX is often loaded to various polymeric or natural hydrogels, including poly(organophosphazene), liposomes, dendrimers, nanoparticles and antibodies [8], or even human serum proteins. Among these serum proteins, serum albumin and transferrin (TRF) have gained most interest due to their potential as drug delivery systems for improving cancer chemotherapy. These proteins are biodegradable, non-toxic, non-immunogenic and exhibit a preferential uptake in tumor tissue [9]. In addition, protein conjugates can accumulate in solid tumors due to the frequently enhanced microvasculature of tumor tissues [10, 11]. This phenomenon has been termed enhanced permeability and retention in relation to tumor targeting (‘EPR-phenomenon’) [12].

Several studies have shown the potential of TRF in the delivery of anticancer drugs to malignant cells that over-express TRF receptors [9]. TRF can covalently be linked to DOX (i.e., DOX-TRF) via a Shiff base which is formed by using glutaraldehyde. This bond was found to be stable at a physiological pH of 7.4 and is not affected by endogenous human enzymes [12]. Inside the cells, DOX is quickly released from the conjugate at pH 5 in endosomes or lysosomes [13].

One of the major factors that causes a pressing need to find a way to deliver anticancer drugs directly to neoplastic cells is the rapid emergence of drug resistant cell populations related to over-expression of ATP-binding cassette (ABC) proteins [14]. P-glycoprotein/MDR1 (P-gp) is a 170-kDa membrane transporter glycoprotein encoded by the *ABCB1* gene. It is known that in patients with breast cancer P-gp expression is a leading cause of resistance to chemotherapy [15]. P-gp is also expressed in other cancer cells and normal tissues under physiological conditions, indicating its major role in the cellular transport of various endogenous substrates and therapeutic agents [16, 17]. Over-expression of P-gp results in an active efflux of anticancer agents from cells and, consequently, a reduction of intracellular drug concentrations and an increased resistance of cancer cells to chemotherapeutic drugs, especially substrate anti-cancer drugs [18, 19]. At present, more than 100 compounds have been identified as P-gp substrates, and one of them is free DOX [20].

Here, we aimed to investigate whether DOX-TRF can impair the role of the P-gp transport system and, if so, what the connection is between this functionality and the cytotoxicity of DOX-TRF. Our experiments were carried out in the absence and presence of the P-gp inhibitor Cyclosporin A (CsA) in chronic erythromyeloblastoid leukemia cells sensitive (K562) and resistant (K562/DOX) to doxorubicin. We found that the investigated cells exhibit different sensitivities to the conjugate. Using flow-cytometry and fluorescence microscopy, we observed alterations in Rhodamine 123 (R123) accumulation in K562 and K562/DOX cells. Moreover, by assessment of *ABCB1* gene expression at the mRNA (RT-PCR assay) and protein (MDR-1 shift assay) levels, we observed different time- and concentration-dependent effects of free DOX and DOX-TRF.

## Materials and methods

### Chemical products

Doxorubicin (DOX) was obtained from Sequoia Research Products (Pangbourne, UK). RPMI 1640 bicarbonate was supplied by Cambrex (Basel, Switzerland). Fetal bovine serum (FBS), penicillin and streptomycin were purchased from PAA (Edinburgh, Scotland). R123, Cyclosporin A and materials for carrying out the conjugation procedure were purchased from Sigma (Darmstadt, Germany). DOX was coupled to TRF using a modified conjugation procedure developed by Berczi et al. [21]. Subsequently, the integrity of the obtained DOX-TRF conjugate was analyzed by mass spectrometry [22]. All other chemicals and solvents were of high analytical grade and were obtained from Sigma (Germany) or POCH S.A. (Gliwice, Poland).

### Cell culture

Chronic erythromyeloblastoid leukemia cells sensitive (K562) and resistant (K562/DOX) to DOX were a kind gift from Prof. J. Robert, Institute Bergonie, France. Cells were grown in 75 cm^2^ tissue culture flasks and cultured in RPMI 1640 bicarbonate medium supplemented with 10 % fetal bovine serum (FBS), 100 units/ml penicillin, and 100 μg/ml streptomycin (PAA, Germany). Cultures were maintained at 37 °C in a humidified atmosphere (5 % CO_2_). K562/DOX cells were grown in the same medium as the parental K562 cells, supplemented with 0.02 μM DOX [23]. Cells were plated in 96-well plates (10,000 cells/well) for cytotoxicity, proliferation and mitochondrial membrane potential assays, and in 6-well plates (100,000 cells/well) for flow cytometry, real-time PCR and MDR-1 protein shift assays. Cells were allowed to grow 24 h before treatment and the cultures were monitored periodically for Mycoplasma contamination.

### Growth inhibition assay

The effect of the DOX-TRF conjugate or free DOX on K562 and K562/DOX cell growth was determined using a MTT dye (3-(4,5-dmethylthiazol-2-yl)-2,5-diphenyltetrazolium bromide) assay in 96-well microtiter plates with flat-bottomed wells (Nunc, Denmark) in a total volume of 100 μl. Cells subcultured at a density of 1x10^4^ were incubated with various concentrations of the DOX-TRF conjugate or free DOX at 37 °C for 72 h. Subsequent to treatment with free DOX or its conjugate, MTT (Sigma) at a final concentration of 0.5 mg/ml was added to each culture well. The dark colored crystals produced by viable cells were solubilized with 30 mM HCl in 2-propanol. The absorbance was determined at 570 nm using a microplate reader (Awareness Technology Inc., USA). The drug concentration required for 50 % growth inhibition (IC_50_) of leukemia cells was determined from the corresponding dose–response curves. In order to examine the effect of Cyclosporin A (CsA) on the cytotoxicity of the DOX-TRF conjugate or free DOX, the cells were pre-incubated with this P-gp inhibitor (30 μM) for 30 min. The CsA dose concentration was evaluated during assessment of its toxicity on the viability of the tested cells.

### Assessment of P-gp activity

For assessment of the activity of P-gp as a transporter across cellular membranes we used Rhodamine 123 (R123). Based on the fact that R123’s intracellular concentration depends on the activity of this transmembrane efflux protein, this fluorescence probe has amply been used for the analysis of P-gp activity. The experiments were carried out according the following procedure. Cells seeded in Petri dishes at a density 1×10^6^ cells/ml were pre-incubated without or with 30 μM CsA for 30 min at 37 °C in culture medium. Then free DOX or DOX-TRF at an IC_50_ concentration was added and the samples were further incubated for 24 h. Subsequently, the culture medium was removed, and the cells were washed twice with PBS and removed from the culture dishes. After centrifugation (300×*g* for 10 min at 4 °C), the cells were re-suspended in ice-cold PBS, and to all samples 50 μl R123 was added to a final concentration of 0.1 M. The cellular uptake of R123 was monitored per 20 min on a flow cytometer (BD Immunocytometry Systems, San Jose, USA), equipped with a 488 nm argon laser and 2 fluorescence detectors: FL1 and FL2. Finally, fluorescence of the analyzed samples after incubation with R123 was evaluated using a fluorescence microscope (Olympus XI-70, Tokyo, Japan) at excitation and emission wavelengths of 497 nm and 520 nm, respectively.

### *ABCB1* gene expression analysis

1x10^5^ cells were seeded per well in a 6-well plate and cultured with an IC_50_ concentration of the examined compounds for 48 h. Total RNA from the cells was extracted using TRI Reagent (Sigma, Germany) according to the manufacturer’s instructions. The RNA concentrations were quantified using an UV spectrophotometer (NanoDrop ND-1000 Spectrophotometer, Thermo Scientific, USA). Then cDNA was synthesized from equal amounts of RNA using a Maxima First Strand cDNA Synthesis Kit for RT-PCR (Thermo Scientific, USA). The primer sequences used were as follows: ERK1 GenBank: ABCB1 forward, 5′-TGGAAACAGTGGCTGTGGGAAG-3′, and reverse, 5′-TCCTGTCCATCAACACTGACCATC-3′; HMBS GenBank: NM_002745 forward, 5′-GGCAATGCGGCTGCAA-3′, and reverse, 5′-GGGTACCCACGCGAATCAC-3′; HPRT1 GenBank: NM_001101 forward, 5′-TGACACTGGCAAAACAATGCA -3′, and reverse, 5′-GGTCCTTTTCACCAGCAAGCT-3′. The expression levels of the different genes were measured by real-time PCR amplification in conjunction with a SYBR Green I Master Mix (Roche) using a LightCycler 480 (Roche, Basel, Switzerland). Finally, threshold cycle numbers (Ct) were used to calculate the expression of each target gene by normalizing to the house-keeping genes hydroxymethylbilane synthase (*HMBS)* and hypoxanthine phosphoribosyltransferase 1 *(HPRT1)*.

### MDR-1 shift assay

Conformational changes in P-gp protein that occur as a result of transporting MDR1 substrates into K562 and K562/DOX cells were evaluated using a R-phycoerythrin (R-PE)-conjugated mouse anti-human P-gp monoclonal antibody (UIC2; Millipore, USA) according to the MDR Shift assay protocol [24]. This assay allowed us to determine to what extent which molecules (DOX or DOX-TRF) act as MDR1 substrates for P-gp. Because UIC2 binding increases in the presence of MDR1 substrates, we utilized this assay for a highly sensitive and specific detection of P-gp expression.

After a 48 h incubation with free DOX or DOX-TRF, cells were collected, washed twice with PBS, and incubated for 15 min at 37 °C with R-PE conjugated anti-human P-gp monoclonal antibody UIC2 (UIC2 PE). After a 15 min incubation at 4 °C, the cells were washed twice with PBS and kept on ice until measurements. As a positive control vinblastine-treated cells were used. Measurements were performed on a flow cytometer using the FL2 channel (BD Immunocytometry Systems, San Jose, USA see above).

### Statistical analyses

Data were expressed as a means ± S.D. Evaluation of the distribution normality of the investigated variables was performed by a Shapiro-Wilk test, and homogeneity of variance was evaluated using Levene’s test. In order to assess statistically significant differences between the studied variables, we performed a multivariate analysis of variance (ANOVA), and Tukey’s test was used for post-hoc analyses [25]. All statistics were calculated using the STATISTICA program version 11 (StatSoft, Tulsa, OK, USA). A *p*-value of <0.05 was considered significant.

## Results

### Increased cytotoxicity of the DOX-TRF conjugate

**Fig. 1:**
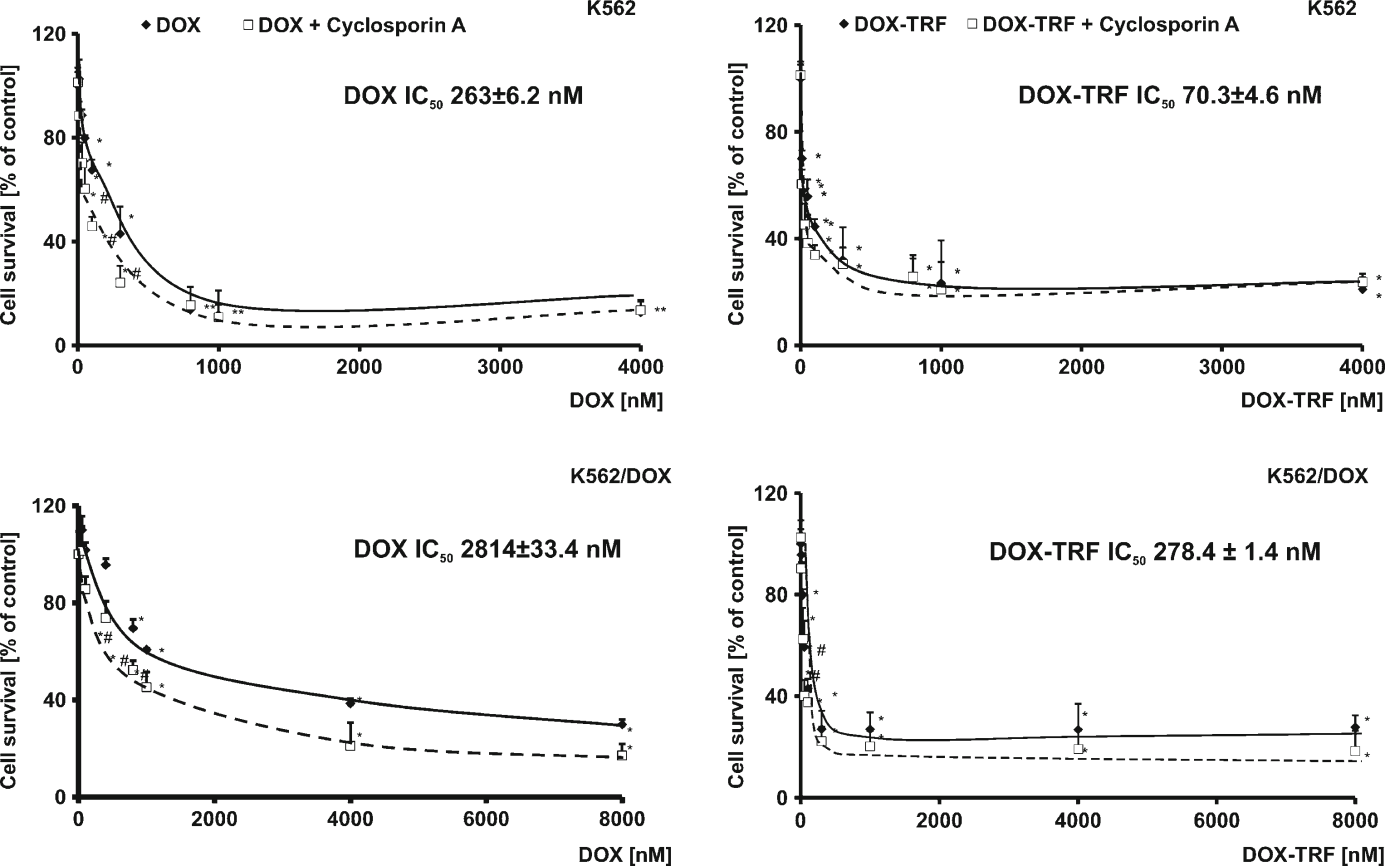
Representative dose–response curves following exposure to DOX and DOX-TRF conjugate of K562 cells sensitive and resistant (K562/DOX) to doxorubicin. The evaluation of cell survival was assessed by MTT assay. ^∗^ *p* < 0.05, significant effects of the examined compounds on the viability of human leukemia cells. ^#^ *p* < 0.05, significant changes between the probes preincubated with Cyclosporin A (CsA). The values are the mean ± SD of four independent experiments

To study the effect of free DOX or DOX conjugated with TRF on sensitive and resistant K562 cells, we carried out a MTT assay. As shown in Fig. 1, a statistically significant reduction in the amount of formazan forming cells in the presence of the examined drugs (DOX or DOX-TRF) was found to be concentration-dependent. However, when the cells were treated with DOX-TRF, the fraction of surviving cells decreased more significantly, both in K562 and K562/DOX cells, compared to those treated with free DOX. The IC_50_ values (indicating the concentration that provides 50 % cell survival after a 72 h incubation period) for the drugs studied were as follows: in K562 cells 263.0 ± 6.2 nM for DOX, 70.3 ± 4.6 nM for DOX-TRF, and in K562/DOX cells 2814.0 ± 33.4 nM for DOX, 278.4 ± 1.4 nM for DOX-TRF. An additional variant of the experiment was the assessment of viability after exposure of the cells for 72 h to CsA at a 0.5–50 μM concentration range. We noticed that the effect of different concentrations of CsA was not statistically significant, and that the survival of cells treated with this P-gp inhibitor did not decrease below 90 % (data not shown). Therefore, we decided to use a 30 μM concentration of CsA, which is in conformity with literature data [26, 27]. In cells pre-incubated with this CsA concentration and, subsequently, treated with free DOX or DOX-TRF, we observed an increased cytotoxicity of both DOX and DOX-TRF (Fig. 1). In K562 and K562/DOX cells treated with free DOX, CsA reduced the survival with about 25 %. A similar reduction in survival was obtained in DOX-TRF treated cells. No statistically significant difference was noted between the two.

### DOX and its conjugate modify the functionality of P-gp

**Fig. 2:**
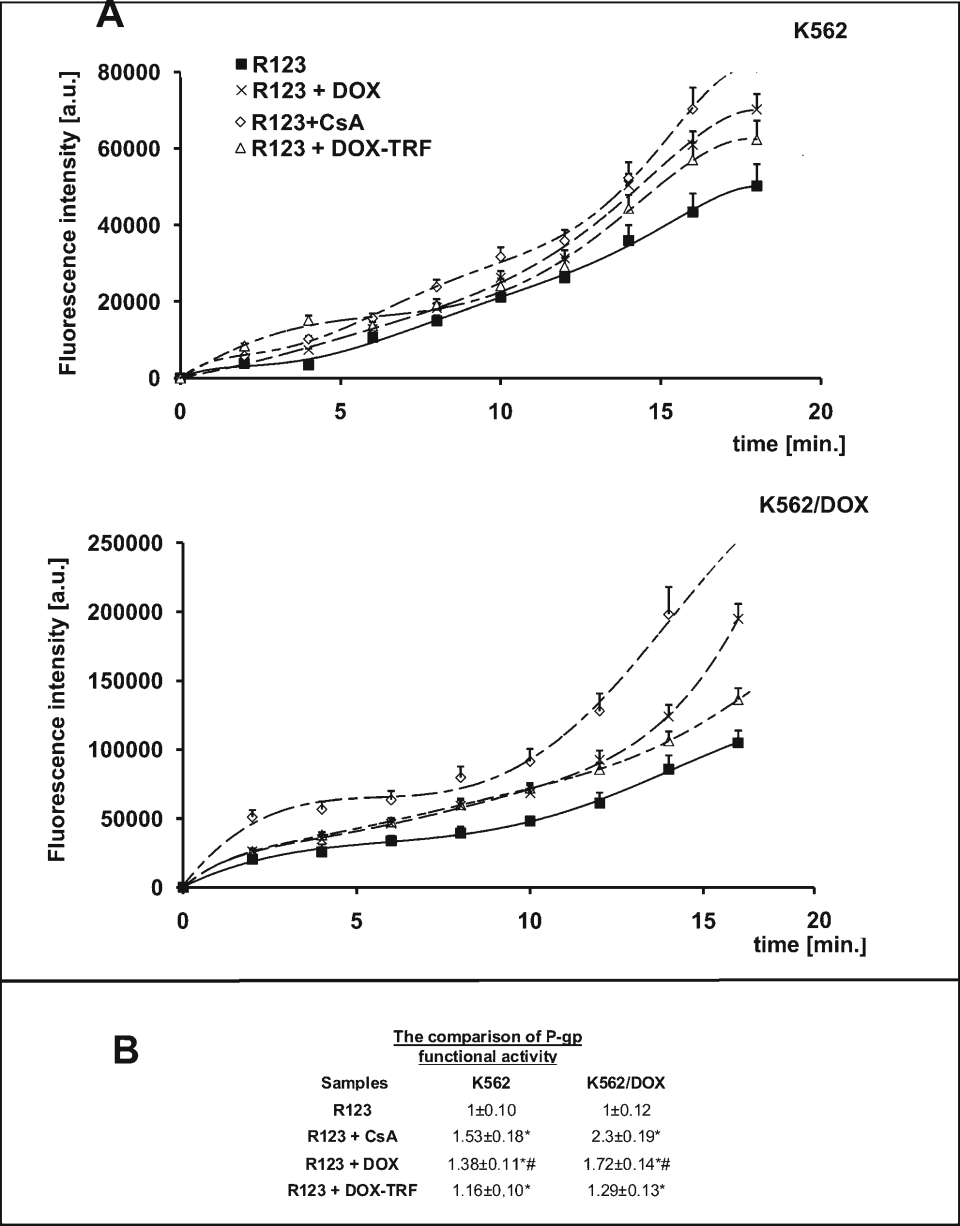
Uptake of R123 in the presence of Cyclosporin A (CsA), DOX or DOX-TRF by K562 sensitive and resistant (K562/DOX) cells as a function of time (A). The values are the means ± SD of three independent experiments. Additionally, on the basis of fluorescence ratios of R123 transport by the examined cell lines, we calculated P-gp functional activities as the slopes of the obtained curves (B). ∗*p* < 0.05, significant differences between P-gp functional activity calculated from the influx of R123 or R123 plus CsA and from R123 plus DOX or DOX-TRF after a 20-min period of monitoring the transport of fluorescence probe to the cells. +*p* < 0.05, significant differences between positive control samples (cells pre-incubated with CsA) and cells treated with DOX or DOX-TRF. #*p* < 0.05, significant differences between cells treated with free DOX or DOX-TRF

**Fig. 3:**
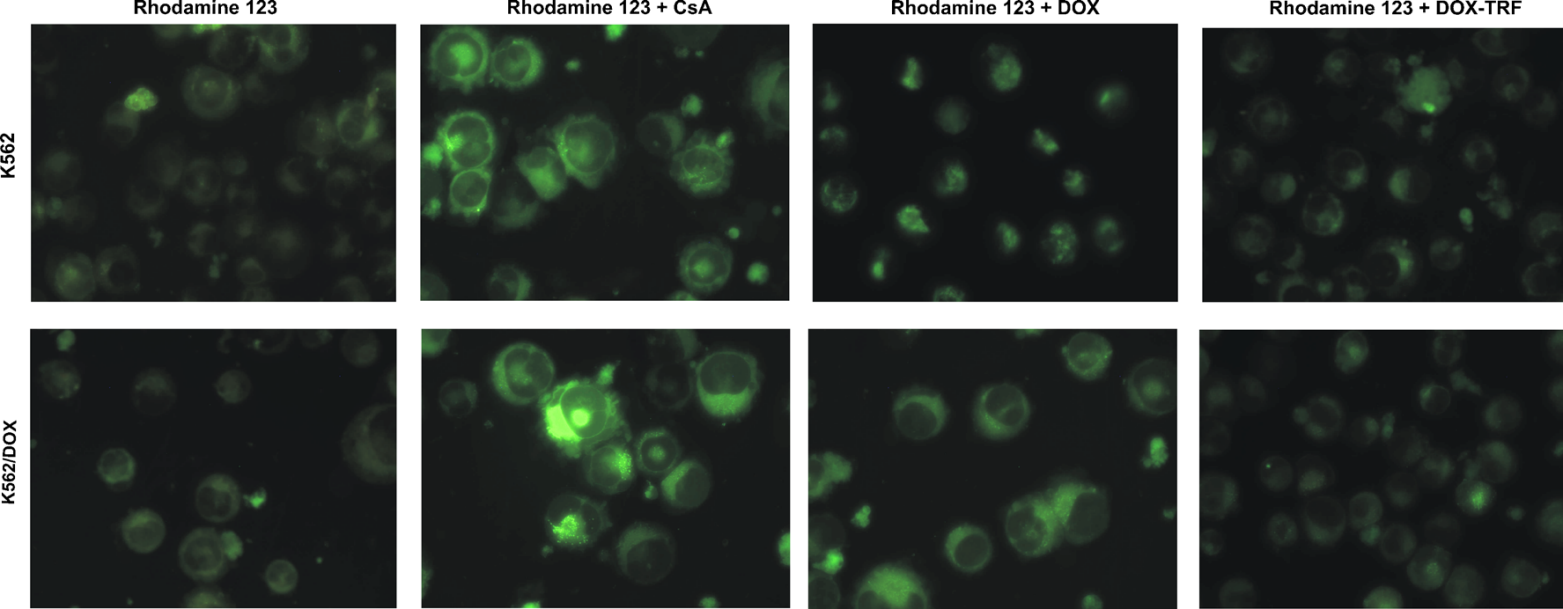
Intracellular R123 accumulation in K562 cells sensitive and resistant (K562/DOX) to doxorubicin. The cells were incubated with IC_50_ concentrations of free DOX or DOX-TRF for 24 h. In the experiments with Cyclosporin A (CsA) the cells were preincubated for 20 min at 37 °C and then incubated with drugs. Fluorescence was monitored using an Olympus IX70 microscope (Japan), magnification 400×

Next, we set out to examine the effect of free DOX and DOX-TRF on P-gp activity by measuring the intracellular accumulation of Rhodamine 123 (R123). The results obtained were compared to those of positive controls, i.e., CsA pre-treated cells. Based on the curves presented in Fig. 2a, fluorescence ratios were calculated for each tested probe. A markedly higher accumulation of fluorescence probes was observed in K562/DOX cells, even though these cells are resistant to DOX (Fig. 2b). In these cells, free DOX was found to increase the R123 cellular influx (1.72 ± 0.14) in a much more noticeable way than DOX-TRF (1.29 ± 0.13). The relative assessment of P-gp activity obtained from the cytometric analysis in DOX-sensitive K562 cells confirmed that both free DOX and its conjugate modify the functionality of P-gp. We found that the investigated compounds can increase the accumulation of R123 by factors of 1.38 and 1.16, respectively, compared to control samples without drugs and CsA pre-treatment. These results, obtained by flow cytometry, were subsequently confirmed by fluorescence microscopy (Fig. 3). We noticed a maximal R123 accumulation in K562/DOX cells pre-treated with CsA. However, when we compared the fluorescence intensity in cells incubated with free DOX or DOX-TRF, we observed a higher R123 accumulation in cells treated with the free drug.

### DOX increases *ABCB1* transcription in K562 and K562/DOX cells

**Fig. 4:**
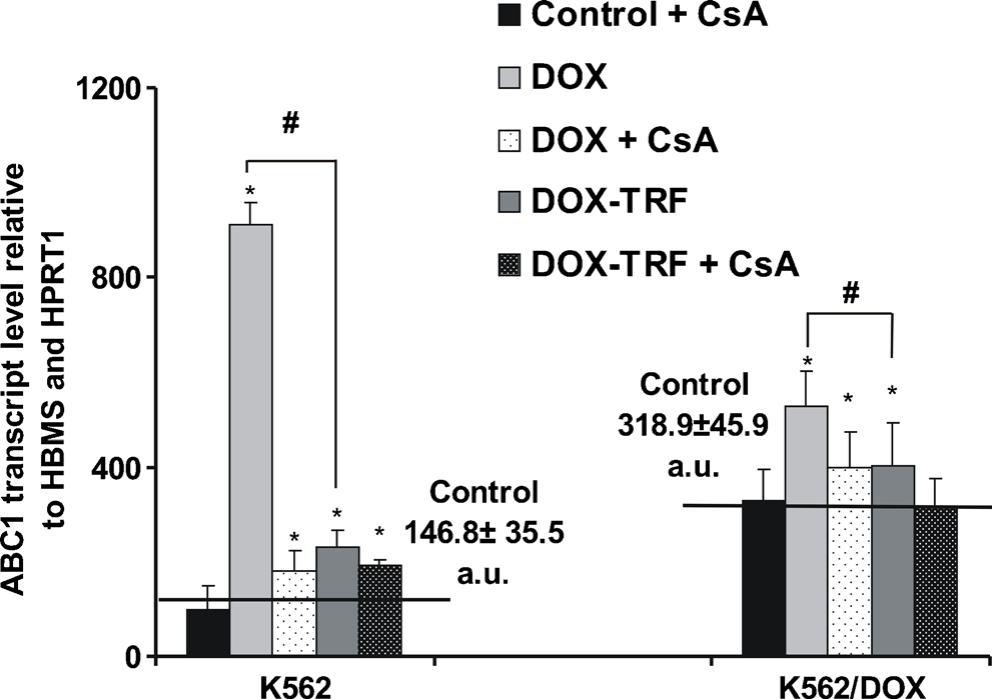
ABCB1 transcript levels (relative to housekeeping genes) in K562 sensitive and resistant (K562/DOX) cells exposed to free DOX or DOX-TRF for 48 h. Asterisks refer to significant differences (*p < 0.05, *n* = 3) in expression levels in cells treated with the investigated compounds compared to untreated cells, whereas hashes (#*p* < 0.05, *n* = 3) relate to significant differences observed between the cells treated with DOX or DOX-TRF

To assess the role of free DOX and DOX-TRF in P-gp expression, we measured *ABCB1* transcript levels (relative to *HBMS* and *HPRT1*) in parental K562 cells and its resistant derivative K562/DOX. RT-PCR analyses revealed that the mRNA expression level was higher in control, untreated K562/DOX cells (318.9) than in K562 sensitive cells (146.8) (Fig. 4). When cells were treated with free DOX, we observed a statistically higher *ABCB1* transcript level (#) compared to cells incubated with DOX-TRF. Free DOX induced an increase in *ABCB1* transcription of 6.2 and 1.7 times in K562 and K562/DOX cells, respectively, whereas the same cells treated with DOX-TRF showed increases in *ABCB1* mRNA expression levels of 1.6 and 1.3 times, respectively. As shown in Fig. 4, pre-incubation of the cells with CsA decreased the *ABCB1* transcript levels in both investigated cell types treated with either free DOX or TRF-conjugated DOX.

### Changes in P-gp expression after DOX or DOX-TRF treatment

**Fig. 5:**
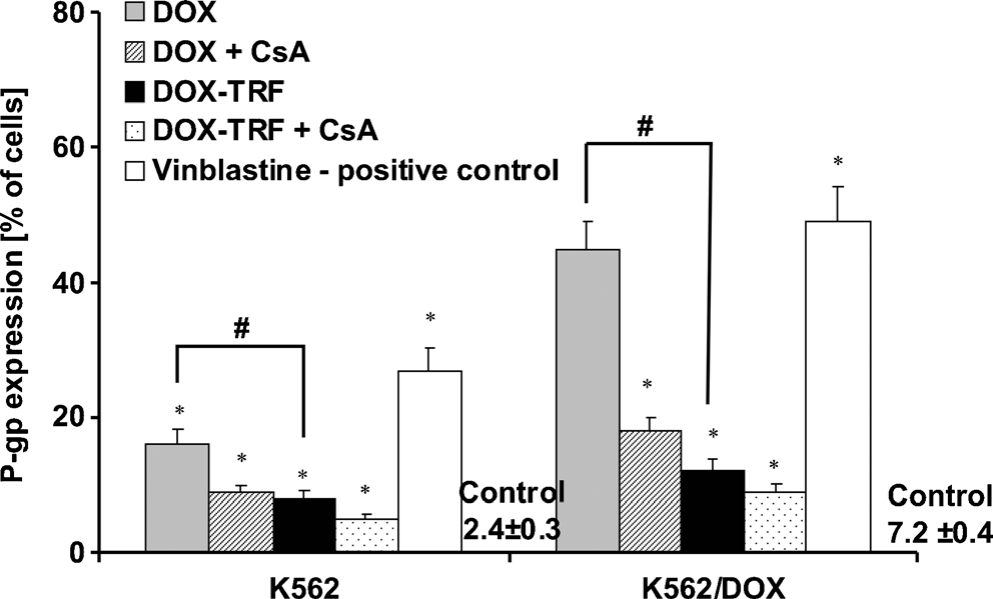
Analysis of P-gp expression in K562 cells resistant and sensitive (K562/DOX) to doxorubicin. Results are presented as means ± S.D. of four experiments. (∗) Significant differences between drug-treated and control, untreated cells, *p* <0.05. (#) significant differences between cells treated with free DOX or DOX-TRF, *p* <0.05

In Fig. 5 changes in P-gp expression are presented after treatment of the cells with free DOX or DOX-TRF. Using monoclonal antibody UIC2 (see section 2), we detected a 3 times higher level of P-gp in K562/DOX cells (7.2 %) than in the parental cell line K562 (2.4 %). In cells exposed to the examined compounds, the highest P-gp changes were found in DOX-treated K562/DOX cells (30 % increase relative to control, untreated cells). Also, DOX-TRF was found to provoke an increase in P-gp change in both cell lines tested, but to a significantly (#) lesser extent than the free drug. Pre-treatment of the cells with CsA indicated that free DOX causes higher changes in P-gp expression than the DOX-TRF conjugate. Vinblastine, which we used as a positive control, confirmed that the expression of P-gp was 2.6 times higher in the K562 cell line resistant to DOX compared to the parental line.

## Discussion

The objective of this study was to test whether the application of a doxorubicin-transferrin (DOX-TRF) conjugate can effectively overcome P-glycoprotein (P-gp) related drug resistance and, thus, improve the efficacy of anticancer therapy. Therefore, we compared the activity, transcription and expression of P-gp in erythromyeloblastoid cells treated with free DOX or DOX-TRF. Our experiments, aimed to assess these various parameters, were carried out in parallel with an evaluation of the cytotoxicity of the investigated anticancer compounds. The cell-killing effects of free DOX and the DOX-TRF conjugate were also tested in combination with a P-gp inhibitor, Cyclosporin A (CsA). Our findings are in agreement with those of Lo et al. [28], who indicated that in a non-small cell lung cancer cell line (A549) some anthracyclines and their derivatives, like aclarubicin, modulate the functionality of P-gp. We also showed that the combination of free DOX plus CsA was more cytotoxic to the investigated leukemia cells than any of the agents used individually. Moreover, we observed an increase in DOX-TRF conjugate cytotoxicity in K562/DOX cells more significantly than in DOX-sensitive K562 cells. Literature data indicate that conjugation of anticancer drugs with nano-sized delivery systems can be obtained with dendrimers, nanoparticles, micelles [29–31] and plasma proteins. These potential carriers, when conjugated with hydrophobic drugs, can increase their cumulative dose and improve their accumulation in the tumor microenvironment over time [32, 33]. Recently, a linkage of TRF to liposomal DOX containing verapamil (VER), i.e., Tf-L-DOX/VER, has been obtained and shown to be able to overcome multi-drug resistance (MDR) [34]. In addition, targeted liposomal delivery has been shown to increase tumor cell-selective cytotoxicity and to facilitate P-gp bypass and MDR reversal. Wu et al. [34] confirmed that TRF-targeted liposomal delivery of co-encapsulated DOX and VER (L-DOX/VER) can be an effective strategy to selectively target and reverse drug resistance in K562 cells. Moreover, these results showed that Tf-L-DOX/VER exhibited an approximately 5 times lower IC_50_ value than that of non-targeted L-DOX/VER. To test the hypothesis whether the higher toxicity of the DOX-TRF conjugate may be a result of its lower exclusion from cells by P-gp related transport, we used R123, which is a known substrate for P-gp, and evaluated the effect of free DOX and conjugated DOX-TRF on its intracellular transport. An increase in P-gp activity was observed in both sensitive and resistant K562 cells incubated with DOX. Our work also indicates that the DOX-TRF conjugate does not modulate the functionality of P-gp, especially in K562/DOX cells. Our flow cytometric data were in agreement with subsequent microscopic observations of R123 accumulation in cells treated with free DOX or the DOX-TRF conjugate, i.e., after 20 min incubation of the cells with R123 we noted a higher fluorescence intensity in cells incubated with conjugated DOX than in those incubated with free DOX.

As yet, the effects of drugs conjugated with human plasma proteins on P-gp activity are still controversial. The anti-tumor effect of the DOX-TRF conjugate, which is readily internalized into drug-resistant cells, has been attributed to a direct binding to P-gp [35]. As far as we know, a higher activity of P-gp is related to conformational changes that occur upon transport of MDR1 substrates. This notion was underscored by comparing conformational changes that occur after treatment with free DOX or DOX-TRF, i.e., by carrying out a MDR1 shift assay we found that UIC2 antibody binding was drastically increased in the presence of free DOX, whereas DOX-TRF caused less detectable conformational changes in P-gp. Also, CsA pre-treatment appeared to confirm that P-gp is critical for the efflux of both free DOX and DOX-TRF, although with different kinetics and affinities. Interestingly, these results were in keeping with *ABCB1* transcript levels assessed by real-time PCR, performed after 48 h incubation with the investigated compounds. In the past, the impact of ATP-binding cassette transporters such as P-gp in mediating multi-drug resistance in chronic erythromyeloblastoid leukemia cells has been reported [36]. We found that transcription of the multi-drug resistance *ABCB1* gene in K562/DOX cells was about 3-fold higher than that in the parental DOX-sensitive cell line K562. Moreover, we found that free DOX treatment causes a more significant increase in *ABCB1* gene transcription than the DOX-TRF conjugate. In P-gp over-expressing K562 cells the increase of DOX-TRF-induced *ABCB1* transcription was not statistically significant.

In summary, we here show that there is a relationship between an increased cytotoxicity of the DOX-TRF conjugate and its effect on P-gp functionality and expression. In contrast to the DOX-TRF conjugate, free DOX leads to a higher expression of P-gp. The higher cytotoxicity of the DOX-TRF conjugate may be associated with its lower affinity to P-gp. Therefore, we suggest that a longer accumulation of the DOX-TRF conjugate and its higher cytoplasmic concentration may not lead to a higher *ABCB1* gene expression level and/or an increased P-gp mediated efflux.
